# The giant panda is cryptic

**DOI:** 10.1038/s41598-021-00742-4

**Published:** 2021-10-28

**Authors:** Ossi Nokelainen, Nicholas E. Scott-Samuel, Yonggang Nie, Fuwen Wei, Tim Caro

**Affiliations:** 1 Department of Biological and Environmental Science, University of Jyväskylä, P.O. Box 35, 40014 Jyväskylä, Finland; 2 School of Psychological Science, University of Bristol, Bristol, UK; 3 Key Laboratory of Animal Ecology and Conservation Biology, Chinese Academy of Sciences, Beijing, China; 4 Center for Excellence in Animal Evolution and Genetics, Chinese Academy of Sciences, Kunming, China; 5 School of Biological Sciences, University of Bristol, Bristol, BS8 1TQ UK; 6 Center for Population Biology, University of California, 1 Shields Avenue, Davis, CA 95616 USA

**Keywords:** Evolution, Ecology, Behavioural ecology

## Abstract

The giant panda (*Ailuropoda melanoleuca*) is an iconic mammal, but the function of its black-and-white coloration is mysterious. Using photographs of giant pandas taken in the wild and state-of-the-art image analysis, we confirm the counterintuitive hypothesis that their coloration provides camouflage in their natural environment. The black fur blends into dark shades and tree trunks, whereas white fur matches foliage and snow when present, and intermediate pelage tones match rocks and ground. At longer viewing distances giant pandas show high edge disruption that breaks up their outline, and up close they rely more on background matching. The results are consistent across acuity-corrected canine, feline, and human vision models. We also show quantitatively that the species animal-to-background colour matching falls within the range of other species that are widely recognised as cryptic. Thus, their coloration is an adaptation to provide background matching in the visual environment in which they live and simultaneously to afford distance-dependent disruptive coloration, the latter of which constitutes the first computational evidence of this form of protective coloration in mammals.

## Introduction

Most mammals have drab coloration, generally showing brown tones, but there are a small number of well-known exceptions that demand evolutionary explanation^1,2^. One is the giant panda (*Ailuropoda melanoleuca*), an iconic flagship species of conservation biology which is familiar to a great many people, but the function of its black-and-white coloration has proven puzzling. Several hypotheses for its strange appearance have been suggested. These include intraspecific signalling^3,4^, heat management^3^, aposematism^5,6^, and background matching^5,7^, the latter two to avoid predation by tigers (*Panthera tigris*), leopards (*Panthera pardus*) and dholes (*Cuon alpinus*), all of which are suggested to prey on giant pandas, especially young ones^3,4^. To date however, the adaptive significance of its seemingly conspicuous black-and-white coloration remains unresolved.

With respect to camouflage^8^, coloration may facilitate crypsis (i.e., preventing detection), for example through both background matching^9^, whereby an animal’s appearance matches the colour, lightness and pattern of its visual background, and disruptive coloration, whereby an animal’s appearance creates false edges or boundaries hindering recognition of its shape^10^. Caro and colleagues^11^ used a comparative phylogenetic analysis of carnivores to propose that the giant panda has cryptic body pelage (while the black markings around the eyes and on the ears could be used in communication). Using comparative methods to score coloration, they showed that lighter fur colour correlates with the presence of snow across terrestrial carnivore species, making it plausible that white areas of the giant panda’s body are cryptic against the seasonal snowy background characteristic of the species’ habitat^11^. In contrast, dark areas are associated with shade in carnivores and ursids, suggesting these parts of the giant panda’s pelage might keep the animal camouflaged in shaded forested areas^12^. This dual function coloration could be driven by the need of this species to traverse two very different visual ﻿backgrounds (winter and summer habitat) during the course of the year, because their diet prevents them from hibernating as do some other Ursidae^11^. While background matching was implicated in these analyses^11^, disruptive coloration^13,14^ was poorly supported. However, the pelage displays two critical features of disruptive coloration: highly contrasting colour patches intersecting the animal’s outline, and coloration that matches at least some visual elements of the background—maximum disruptive contrast and differential blending, respectively^8,15,16^.

**Figure 1:**
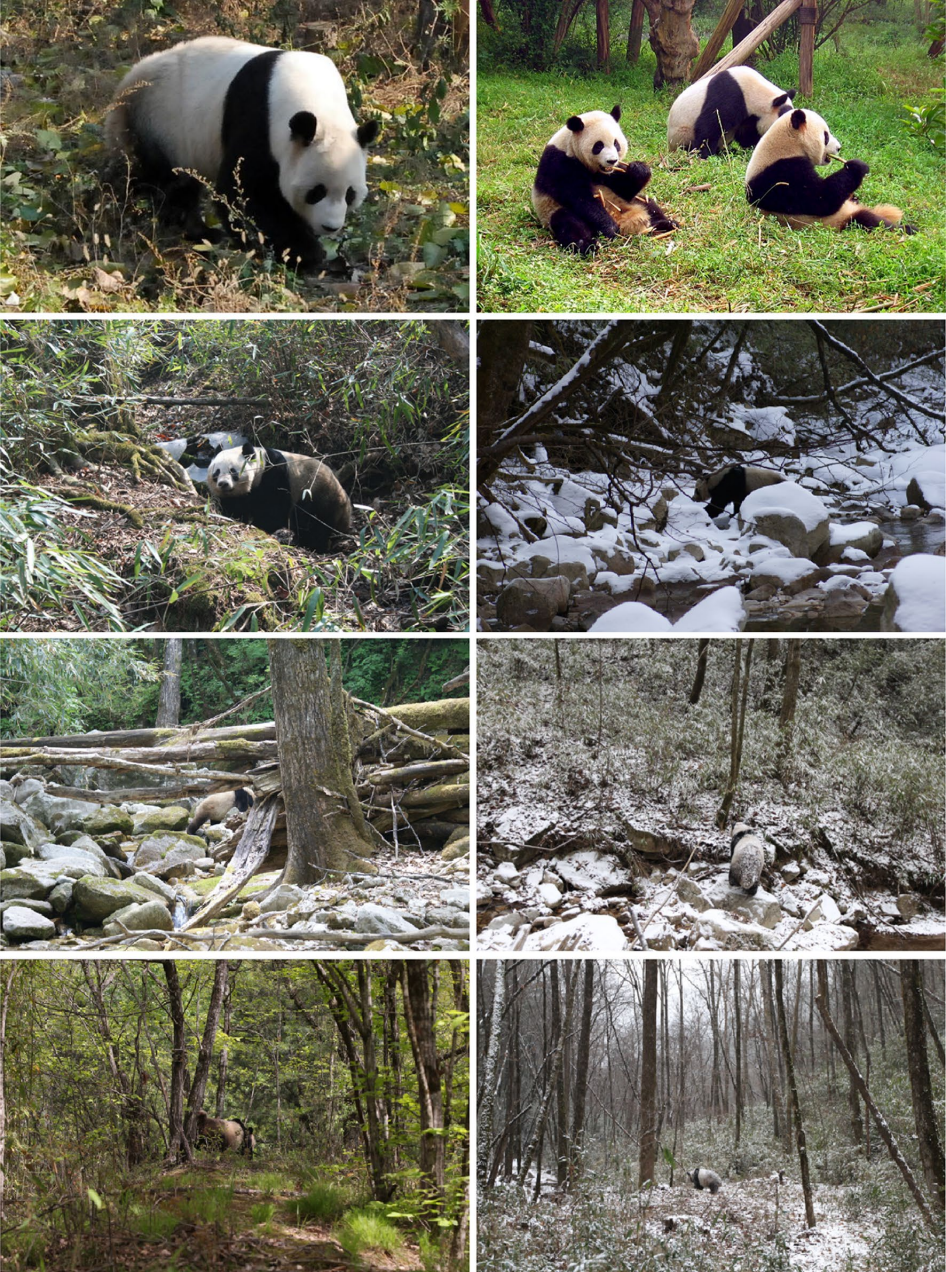
Giant panda coloration and its visual background. First row: animals photographed in a zoo (left Beijing zoo, right Chengdu zoo, Wikimedia commons, the rest in nature by FW), exemplifying how black-and-white coloration stands out when viewed close up and/or against an artificial background. Second row: In its natural habitat, the coloration is less conspicuous even at close range. Third row: the camouflage is more evident at intermediate distances and from various angles, and helps the giant panda to blend into its natural environment. Bottom row: from further away, the black-and-white coloration breaks up the outline of the animal due to increased edge disruption. Note that these figures are meant illustrative—the images are not normalised data.

We explore the hypothesis that the giant panda is cryptic in its natural mountain forest habitat, despite the fact that the species’ black-and-white coloration appears conspicuous when viewed close up (Fig. 1). To achieve this, we examined difficult-to-obtain photographs of 15 giant pandas taken in the wild using the Multispectral Image Calibration and Analysis (MICA) toolbox^17^ and the Quantitative Colour and Pattern Analysis (QCPA) framework^18^, which together provide state-of-the-art image analysis^17–20^, to establish whether or not giant pandas are camouflaged either through background matching or disruptive coloration, or both. We note that the sample size is not large, but fifteen replicates are adequate to address some of the basic questions related to their mysterious appearance from a visual ecology perspective.

**Figure 2:**
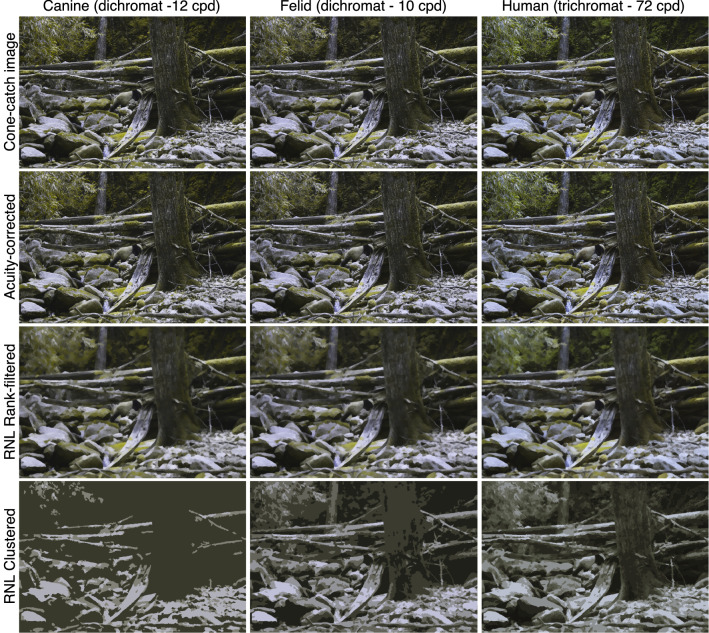
Quantitative colour and pattern analysis (QCPA) was used to investigate the giant panda coloration in its natural habitat. We studied how closely the black-and-white pelages of the giant panda clusters with the visual patchiness of the background. The RNL clustering method uses spatial filtering to determine which visual elements group together in the image. Here, one example of the giant panda images is shown and modelled using dichromatic canine (domestic dog) and felid (cat) vision, and trichromatic human vision. The first row shows images converted to receiver dependent cone catch images, the second row shows acuity-corrected (canine—dog 12 cpd, felid—cat 10 cpd, human 72 cpd,) images, the third row shows RNL rank-filtered images and the fourth row shows RNL-clustered view. These examples were gamma corrected for better image screening.

First, using the QCPA framework and its acuity-corrected cluster analysis (RNL clustering), we studied how closely the black-and-white pelage of the giant panda clusters with the visual patchiness of the background. This approach uses spatial filtering to determine which colour and/or luminance elements cluster together in the image (Fig. 2). We assumed that the pelage would have counterparts (i.e., similar brightness elements) present in the visual background if they were to serve a background matching function. We modelled the image data with dichromatic (canine and felid—predator surrogates) and trichromatic (human—a point of reference) vision systems and their corresponding visual acuities. Second, using Gabor filtering to quantify the ratio of false edges that run orthogonal to an animal’s true outline^19^, we probed whether the giant panda demonstrates disruptive coloration operating as a function of distance. We hypothesised that edge disruption, if it occurs, would operate more easily on animals further away because, as the distance between predator and prey increases, the latter’s outline will become less visible than the highly salient disruptive markings^15,21,22^. At the limit of object detection (e.g., at the maximum distance at which any part of the panda can still be seen by a potential predator), both disruptive markings and the animal’s outline should become undetectable. Finally, to debunk the myth of conspicuousness of giant pandas in their natural environment, a common misconception, we asked whether giant panda coloration matches the background colour variation as assessed by a colour map comparison technique (a component of QCPA) using human vision as a proxy^18^. We compared this similarity-to-background metric in the giant panda to a variety of other species examined objectively along the “crypsis-conspicuousness spectrum”. Our results provide information on how conspicuous black-and-white coloration in animals may provide concealment in the visual environment in which they live and simultaneously afford distance-dependent disruptive coloration.

## Results

### Quantification of background matching: how does the patchiness of the black-and-white coloration blend into the visual environment?

The giant panda pelage patches were significantly associated with visual elements found in their natural habitat (Chi-Square test for independence, χ^2^ = 89.01, df = 10, *p* < 0.001). There were no patches that were unique to the giant panda and not found in the background in terms of luminance. The black patches clustered with shadows and naturally dark tree trunks, white patches clustered with foliage (due to specular light reflecting from the leaves under high irradiance) and snow when present, and midtones clustered with ground and rocks. Although people tend to consider the giant panda as dichotomously black-and-white, we detected an additional visual cluster of off-white fur, which characterizes midtones in terms of luminance (i.e., not being dark or light).

**Figure 3:**
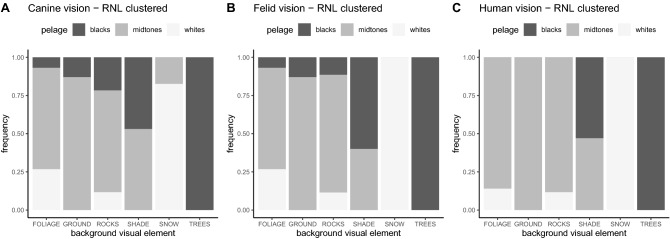
Background matching of the giant panda. The clustering method uses spatial filtering to test which visual elements cluster together in the image. The vision modelling was performed using acuity-corrected cone-catch images. We used three vision models: (**A**) canine vision (dog 12 cpd) and (**B**) felid vision (cat 10 cpd), as the dichromatic predator surrogates, and (**C**) the trichromatic human vision (72 cpd) for the sake of comparison and to provide a point of reference. The clustering results show that the dark elements (e.g., shadows, tree trunks) in the background are grouped with black pelage patches, brightest elements (e.g., snow, bright leaves in foliage and rocks) are grouped together with white pelage patches, whereas elements with midtones (e.g., ground, but also rocks, leaves and shades) are grouped with intermediate off-white pelage patches. The frequency of each subgroup is represented allowing a comparison of the contribution of their proportion to the whole. The frequencies (0–1) are additive to 100% within the visual background comparison group.

More specifically, background visual elements (i.e., shade, trees, ground, snow, foliage, rocks) clustered between the black, midtones and white colours of giant pandas’ pelage as follows (Fig. 3). On average across the three visual models, the black patches were associated with dark tree trunks (57.6%) and shadows (30.7%), but also with ground (3.8%), rocks (3.8%) and foliage (3.8%). The white patches were grouped with snow (on average 60.0% of the images, but always when snow was present), waxy bright foliage (30.0%) and rocks (10.0%). The midtones were clustered with ground (35.8%), foliage (28.2%), shady background areas (17.9%), and also with rocks (17.9%).

**Figure 4:**
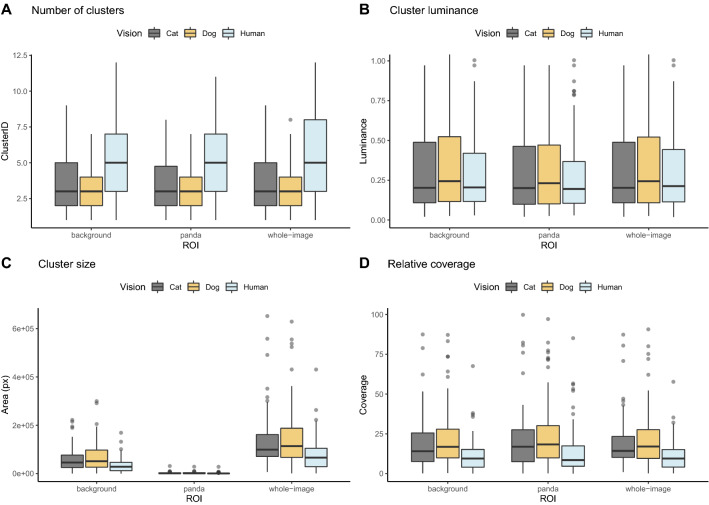
Background matching of the giant panda. The figure shows the relationship between the measured region of interest (ROI) and vision model used. Clustering results are presented for (**A**) number of clusters formed, (**B**) cluster luminance distribution, (**C**) cluster size measured in pixels (thus, de facto area is arbitrary and represents between group differences), and (**D**) the relative coverage of cluster size within the measured ROI (background, panda, whole image). The clustering method uses spatial filtering to test which visual elements cluster together in the image. The vision modelling was performed using acuity-corrected cone-catch images (canine vision—dog 12 cpd and felid vision—cat 10 cpd, the dichromatic predator surrogates, and the trichromatic human vision 72 cpd).

QCPA showed similar clustering results of background matching for ROI comparisons (i.e., regions of interest: the panda, the background, the entire image) as regards number of visual clusters formed, cluster luminance and relative patch coverage (Fig. 4, Tables S3–S7). Also, the majority of Visual Contrast Analysis (VCA) metrics overlapped when comparing values across the giant panda, the background and the entire image (Fig. S3). However, two variables from dichromatic felid (cat) and canine (dog) vision models showed a marked difference from higher human vision model values: weighted mean of RNL pattern contrast (VCA:MSL) and weighted standard deviation of RNL luminance pattern contrast (VCA:sSL). The comparison of clustering results between seasons (summer, winter) did not show marked differences (Fig. S4).

### Quantification of disruptive coloration

**Figure 5:**
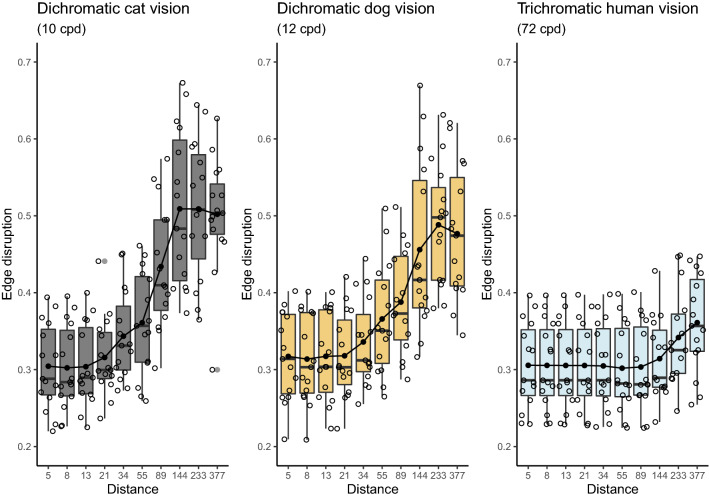
Giant panda edge disruption. The method uses Gabor filtering to quantify the ratio of false edges that run orthogonal to the animal’s true outline. Edge disruption was modelled through three visual models and their respective acuities: dichromatic felid (cat—10 cpd) and canine (dog—12 cpd), as well as trichromatic human vision (72 cpd) for comparison. The edge disruption increases as a function of distance before levelling out at longer distances. The boxplot shows minimum and maximum (whiskers), median horizontal line and the interquartile range of the simulated data.

We found that giant panda edge disruption was poor at close proximity (Figs. 5 and S5), compared to edge disruption that was measured further away. The minimum (or poorest) edge disruption was observed at approximately 12 m (at 8 relative body lengths distance). We speculate that maximal edge disruption should be close to 50 percent, because beyond this the ‘false edges’ become predominant, leading to the same ‘problem’ of having an outline. At a distance of approximately 50 m (approximated as 34 relative giant panda body lengths), however, edge disruption increased steeply for dichromats, after which it levelled out at even longer distances (edge disruption, distance by vision model interaction, F_2,430_ = 39.77, *p* < 0.001, Fig. 4). Significantly, canine and felid vision systems had steeper edge disruption by distance dependency than human vision, which may suggest that edge disruption works particularly well against visual systems with lower acuity.

### Debunking the myth of conspicuousness: how similar is the colour of the giant panda to its natural habitat and in comparison to other species?

**Figure 6:**
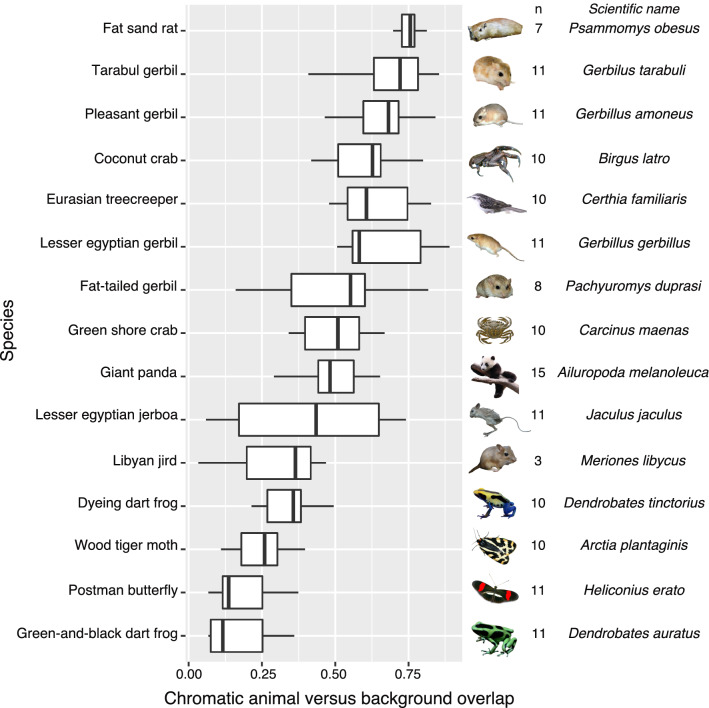
Similarity to background measured as overlap in colour space. The chromaticity diagram using human vision shows that 50% of all colours within images were perceptually similar between the giant panda and its background. The least similar species to their respective backgrounds are those usually considered as being aposematic, whereas the other end of the spectrum consists of the camouflaged animals. The giant panda falls at the middle of this ‘conspicuity spectrum’, and among other species that are considered as camouflaged, suggesting that giant panda pelage coloration does not stand out against the background more than a concealed species would. All inset pictures: Wikimedia commons open access license (CC BY-SA 3.0).

The giant panda is apparently conspicuous to the human eye when seen at short viewing distances. In its natural habitat, however, on average 50% of all colours within an image are perceptually similar to human vision between a giant panda and its visual background (i.e., similarity-to-background chromatic overlap, n = 15 images, s.d. = 0.1) as judged from colour space histograms (Figs. 6 and S2). Colour space overlap comparisons of 15 species, whose adaptive significance has been demonstrated quantitatively, confirmed the expectation that the highest degree of average chromatic similarity-to-background was found among well-camouflaged desert rodents, whereas the lowest degree of average chromatic similarity was found among aposematic poison frogs (similarity index by species, F_14,134_ = 18.99, *p* < 0.001). Giant pandas fall between jerboas and shore crabs, species classically assumed to be cryptic in the field (Fig. 6). These results debunk the common misconception that the giant panda would be conspicuous to human vision in their natural habitat.

## Discussion

We have shown that the iconic black-and-white coloration of the giant panda is cryptic in its natural environment. Furthermore, the giant panda shows distance-dependent disruptive coloration^21,23^. Although disruptive coloration has been discussed in relation to other mammalian taxa before^24,25^, this is the first time to our knowledge that disruptive coloration has been shown to occur in mammals using a quantitative set of computational image analysis techniques.

First, we demonstrated that giant panda fur pelage shares luminance appearance (i.e., has similar counterparts) with its natural visual environment. The black pelage patches blend with dark shadows and tree trunks, thus providing concealment against darker elements in the visual scene whereas white patches match foliage and snow (when present). The brightness variation of the background highlights explains why white is associated with foliage, which are snow-covered during winter and have a waxy outer cuticle (causing specular lighting) during summer. Thus, it seems plausible that white patches can provide concealment in well illuminated forest gaps even without snow, when small gaps in the canopy allow bright light to penetrate the forest. In addition, infrequent pelage midtones match the ground and rocks. We argue that these midtones enhance background matching in the giant panda by providing an intermediate coloration, which bridges the gap between the very dark and very light visual elements in its habitat. Plausibly, the midtones allow more effective background matching than if the panda were just black-and-white. This provides adaptive insight into the brown-and-white coloration of Sichuan and Qinling giant panda individuals, because similar (brown) midtones replace black pelage on individuals in these populations. In the natural visual environment, which mostly consists of bright illuminant small gaps, larger gaps and partial woodland shade^12^, the contrasting black-and-white coloration thus facilitates background matching. The results obtained from both dichromatic and trichromatic vision models were consistent, although two metrics (weighted mean of RNL pattern contrast and weighted standard deviation of RNL luminance pattern contrast) suggest that dichromats may perceive the giant panda’s coloration more uniformly than trichromats. Overall, our data confirm previously reported findings (based on an independent and different comparative methodology) that giant panda coloration matches its background^11^.

Second, using Gabor filtering^19^ and three vision models, we have shown that the giant panda demonstrates disruptive coloration operating as a function of distance: edge disruption is greater at longer distances in comparison with shorter viewing distances, where there may be more reliance on background matching. The latter would always be expected to operate as a function of distance: when an animal is far enough away both it and its background will be blurred, at which point background matching will obtain regardless of the spatial structure of individual colours. In this context, disruptive coloration is particularly interesting because there appears to be an intermediate stage when the spatial structure of the disruptive elements can be resolved even when the target shape cannot. Interestingly, we found that dichromatic visual systems with lower visual acuity than human vision showed steeper edge disruption by distance. Disruptive coloration may therefore work particularly well against visual systems with low acuity, especially when viewed over longer distances. Our results support the idea that edge disruption would be easier to detect on animals further away and with low visual acuity, because as the distance between predator and prey increases, the latter’s outline becomes less visible than the disruptive markings. Plausibly, disruption of the giant panda’s outline can no longer be resolved by the visual system of a potential predator at some ecologically relevant viewing distances, because spatial visual acuity is often poor in large carnivores^26^, and leads to distance dependent pattern blending^21,23^. For example, spotted hyaenas (*Crocuta crocuta*) cannot resolve the widest stripes on a plains zebra (*Equus burchelli*) beyond 50 m distance under lighting conditions when they hunt^26^. At large viewing distances, we believe that both the disruptive markings and the animal’s outline will be undetectable^21,27,28^. We suspect that the giant panda’s coloration shows features of both maximum disruptive coloration and differential blending depending on who views them and from which distance^8,15,16^.

Third, our data busted the myth of conspicuousness of giant pandas. Although at close distances, as in captive settings, the species appears conspicuous to humans, giant pandas share more than half of their colours with their natural habitat. In terms of a similarity-to-background index (i.e., chromatic animal versus background overlap) using the colour map technique, the giant panda groups together with several species widely considered as camouflaged.

Any appearance in nature is dependent on viewing conditions and perceptual sensitivities, as well as the visual acuity of the observer. In light environments which mostly consist of bright illuminant small gaps, larger gaps and partial woodland shade^12^, contrasting black-and-white coloration can serve as both background matching and disruptive coloration, and this possibility should be explored further in other animals with conspicuous black-and-white markings. It is plausible though that the black-and-white coloration is conspicuous close up, and we cannot therefore discount the possibility that visual signalling, whether for conspecifics or heterospecifics, would be important for the giant panda^29^, a conclusion reached by Caro and colleagues^11^ for the black eye and ear markings of this species. In summary, the giant panda uses black-and-white pelage as a form of crypsis to avoid detection in its natural habitat and its apparent conspicuousness to the human eye is an artefact of the short viewing distances found in photography and zoos.

## Methods

### Photographs of the giant pandas in their natural habitat

The giant panda lives in fragmented, humid and remote mountain forests in Sichuan, Shaanxi and Gansu Province in south-central China, which makes it difficult to obtain data on this endangered animal in its natural habitat^11^. Fifteen encounters (assumed to be of different individuals) were photographed from 2007 to 2014 by FW in Sanguanmiao Field Station, Foping Nature Reserve, Shaanxi Province. The photographic data were collected from a narrow geographic range, but we have no reason to expect that our results would be limited to only within our sampling region.

Animals were photographed from various distances (estimated to be approximately 5–150 m) as they were encountered in their environment. The timing of the photographs varied across seasons: some (7) were photographed when there was snow on the ground whereas others (8) were photographed in summer conditions. The environment in these areas comprises several distinctive visual elements such as rocks, ground substrate, small streams, primary forest tree trunks, secondary growth shrubs (including bamboo), foliage and a mixture of alternating bright sunlit spots and dark shady areas. The forest geometry plausibly corresponds to light profile of forest shade with small light gaps^12^. As it was not feasible to explore the irradiance profiles of these different forest patches here, we used standard D65 illumination in the photographs as a proxy. For details of photography and using the micaToolbox as well as QCPA see appendices (Table S1).

### Image analysis techniques

We examined photographs of 15 giant pandas using the Multispectral Image Calibration and Analysis (MICA) toolbox in Image J (version 1.52 k/Java 1.8.0_172 (64-bit)^17^ and its extension, the Quantitative Colour and Pattern Analysis (QCPA) framework^18^—micaToolbox version 15.3.2019. Photographs were processed in digital negative format (.dng) and we used a Canon EOS400D with kit specifications (18–55 mm lens) in quantitative colour and pattern analysis framework (for details see Table S1). Each image was converted into multispectral image stacks as follows. The photographs’ white balance was adjusted through the MICA toolbox plugin with ImageJ software and was normalised to 7 percent black using the giant panda black (dark tones) and 63 percent white reflectance using the white (light tones) pelage patches and 97 percent snow reflectance as proxies (Table S1). The reflectance values of the pelages were confirmed prior to analyses. For this, two captive giant pandas were photographed in Ähtäri Zoo, Finland, next to calibrated photographic standards (Fig. S1). Note that the exact values of fur reflectance are not crucial, but it is important to normalise the dynamic light range across the photographs^30^. We used panda size to standardise scale across the photographs: length was used as ‘a measuring stick’ to approximate relative distance in the edge disruption analysis (see below). As regions of interest (ROIs), the entire animal outline (animal) and the remaining surrounding visual environment excluding the animal (background) were selected.

#### Quantification of background matching

We tested how closely the pelage patches of the giant panda clusters with the visual patchiness of the background. The RNL clustering method uses spatial filtering to determine which luminance elements group together in the image (i.e., the method clusters pixels according to their perceived colour or luminance contrast—our approach for this question takes account of luminance only, Table S1—as measured in Euclidian distance between pixels in the RNL colour space). For details see^18^. Acuity-corrected cone catch images were used without distance scaling for three vision models: domestic dog and cat, and human vision. Details are reported in Table S1. The obtained clusters were counted as dummy variables (i.e., dichotomous: true or false) with respect to whether or not each pelage grouped with specified background visual features; associations between giant panda pelage patches and the ground, leaves, rocks, shades, snow and tree trunks were determined using relative frequencies (i.e., number of associations divided by the total number of samples). We also used the QCPA’s Visual Contrast Analysis (VCA) for detailed luminance analysis (Table S2), and to confirm the validity of human vision modelled data in comparison to dichromatic vision systems (domestic dog and cat) with the following visual acuities: dog 12 cpd^31^, cat 10 cpd^32,33^, and human 72 cycles per degree—cpd^27^.

#### Quantification of disruptive coloration

We tested whether free-living giant pandas show disruptive coloration operating as a function of distance, using Gabor filtering of achromatic edge disruption which quantifies the ratio of false edges that run orthogonal to an animal’s true outline^19^. This approach allowed us to test edge disruption using only the luminance channel. The pictures were first standardised to fixed pixels per distance ratio. The distance was modelled as relative body lengths (the panda body length was set as 1, which corresponds to approximately 1.5 m). We modelled the distance to target using the acuity view tool in the toolbox^18^ with Fibonacci sequence increments to obtain exponential growth for longer distances (starting at 5). A GabRat Sigma value of 3 was used^19^. We modelled edge disruption through all three vision systems explored here (cat, dog, human) and used these as a proxy for the giant panda’s would-be predators^26,34^, because the latter’s exact spatial acuities remain uncertain.

#### Exposing the myth of conspicuousness

We tested whether giant panda coloration matches natural background colour variation as judged by a colour map comparison technique^18^. These colour maps can be considered as a non-parametric histogram representation of the spectral variation captured in any image region using receptor noise limited colour space and acuity correction. The colour map tool in QCPA quantifies chromatic contrast as ΔS in the chromatic Receptor Noise Limited (RNL) space^18^. As such, it categorically ignores luminance^35^ and exclusively quantifies chromatic similarity. The method allows the user to estimate the overlap of estimated colour perception between parts of images. As we wanted to question and demystify the idea of wild giant pandas being conspicuousness to us, we used human vision as a proxy. Images were first converted to normalised multispectral images, as above, and then converted to human cone catch images in D65 standard illuminant. The colour space overlap value (range 0–1, i.e. 0 to 100% similarity) was determined by opposing the human mw:lw channel against the (lw + mw):sw channel, and thus represents the view through a trichromatic visual system. The overlap was considered as a similarity-to-background index where higher values indicate higher similarity between the animal and its background (Fig. S2).

As the QCPA’s colour map method is relatively new and has not been widely used yet, we performed additional comparisons with other species to cross-reference where this similarity-to-background index fell in comparison to the colour patterns of species whose adaptive significance has been demonstrated quantitatively. For this, we used the following species for which we had access to multispectral images (all these are broadly considered as camouflaged): seven species of Sahara-Sahel desert rodents^36^: (*Gerbillus amoenus*, *G. gerbillus*, *G. tarabuli*, *Jaculus hirtipes*, *Meriones libycus*, *Pachyuromys duprasi*, and *Psammomys obesus*), the coconut crab (*Birgus latro*)^37^, Eurasian treecreeper (*Certhia familiaris*; Nokelainen et al. n.d.) and green shore crab (*Carcinus maenas*)^38^. In addition, ON photographed four ‘vividly-coloured’ species (all considered as aposematic): the postman butterfly (*Heliconius erato*), the green-and-black dart frog (*Dendrobates auratus*), a dyeing dart frog (*Dendrobates tinctorius*) and the wood tiger moth (*Arctia plantaginis*). The same QCPA colour map procedure as above was applied to all of these species in order to create colour maps for the reference species in the same way as for giant pandas (Table S1).

### Statistical analysis

First, we used acuity-corrected cluster analysis to determine how well the visual patchiness of the pandas and backgrounds match. Following the cluster analysis, the number of shared visual features were counted: the relative frequencies were collated according to whether the clusters were unique to the giant panda and not found in the background, specific to the background only, or shared between the animal and the background. In addition, we determined which visual elements of the background (i.e., rocks, foliage, trunks, ground, snow, shadows) cluster with differently coloured body parts of giant pandas. The non-independency of background elements and giant panda colour patches was tested with Pearson’s Chi-squared test. The number of visual clusters formed, cluster luminance, cluster size and their relative coverage were tested in a lmer-model (Tables S3–S7). Second, we used Gabor filtering^19^ to quantify the ratio of false edges that run orthogonal to an animal’s true outline. The edge disruption index was used as a dependent variable and the modelled distance and its interaction with the vision model were used as explanatory variables in a lmer-model (lme4-package in r) with ID set as a random factor to control data structure (Table S8). Third, to test whether giant pandas are camouflaged either through background matching, disruptive coloration or both, we used colour space overlap values as a similarity-to-background index and applied this as the dependent variable in a linear model with reference species as the independent variable. Analyses were performed in R studio (R version 3.6.1/2019-07-05).

## Supplementary Information

Supplementary Information 1.
