# Supplementary material for: The giant panda is cryptic
Source: Sci Rep. 2021 Oct 28;11:21287. doi: 10.1038/s41598-021-00742-4 (PMC8553760; doi:10.1038/s41598-021-00742-4)
Supplement: Supplementary file 1 — Supplementary Information 1. [file 41598_2021_742_MOESM1_ESM.pdf]

Supplementary material for Nokelainen et al. 2021 Scientific Reports

Title: The giant panda is cryptic

Authors: Ossi Nokelainen[1*], Nicholas E. Scott-Samuel[2], Yonggang Nie[3,4], Fuwen Wei[3,4], and Tim Caro[5,6*]

[*] Author(s) for correspondence: ossi.nokelainen@jyu.fi, tmcaro@ucdavis.edu

Addresses:[1]Department of Biological and Environmental Science, University of Jyväskylä, P.O. Box 35, 40014 University of Jyväskylä, Finland

[2]School of Psychological Science, University of Bristol, Bristol, UK

[3]Key Laboratory of Animal Ecology and Conservation Biology, Institute of Zoology, Chinese Academy of Sciences, Beijing, China

[4]Center for Excellence in Animal Evolution and Genetics, Chinese Academy of Sciences, Kunming 650223, China

[5]School of Biological Sciences, University of Bristol, Bristol BS8 1TQ, UK

[6]Center for Population Biology, University of California, 1 Shields Avenue, Davis, CA 95616, USA

Contents: Page

Table S1 (methods) 2

Table S2 (methods) 6

Table S3-8 (results) 8-13

Figure S1-5 (results) 14-18

Supplementary references 19

Table S1: Details on photography and QCPA framework methods.

Photography methodology using the micaToolbox and QCPA

micaToolbox version and ImageJ version: We examined photographs of 15 free-living giant pandas using Multispectral Image Calibration and Analysis (MICA) toolbox in Image J (version 1.52k / Java 1.8.0_172 (64-bit) (Troscianko and Stevens 2015) and its extension Quantitative Colour and Pattern Analysis (QCPA) framework (mica version 15.3.2019) (van den Berg et al. 2020).

Equipment: The photographs were taken in the wild using the cameras available at time during the expeditions to mountains. Ideally, cameras should have been kept the same, but this was not possible. The kits were: Canon EOS 1D-mk4 + Canon EF16-35mm f/2.8L II USM; Canon EOS 350D + Canon 18-55mm f3.5-5.6 II; Sony DSC DSLR-A100 + Minolta/Sony AF 75-300mm f/4.5-5.6, and; Sony DSC HCR-42. The photographs were taken from varied distances against natural backgrounds in the wild.

Camera settings: The settings were allowed to vary in order to achieve the best exposure under variable natural lighting conditions. The white balance was normalized in Image J using the approximate natural grey standards in the visual scene (as grey standards, see further).

Image file type: Photographs were taken in jpg format and processed further in digital negative format (dng) to control for potential camera-specific differences in the file type. Ideally, one should opt for uncompressed raw file format, but this was not feasible in our case. We opted for the use of digital negative as an alternative solution, which is a standardised format of saving photographic data in lossless format. Noteworthily, this could be a solution for researchers dealing with virtual museum data from different collections and with no access to the device through which the photographs were taken.

Camera calibration: We used a Canon EOS400D with kit specifications (18-55 mm lens), because this was the closest match available to the camera equipment used.

Illuminant: All photographs were taken under natural light conditions. The photographs were taken in mostly cloudy but bright daylight conditions allowing the light to scatter through the forest geometry. Thus, in the modelling the D65 daylight spectrum was used.

Grey standards: The photographs' white balance was adjusted through the MICA toolbox plugin with ImageJ software, and were normalised to 7 percent black using the giant panda black (dark tones) and 63 percent white reflectance using the white (light tones) pelage patches as proxies. When snow was present (in approximately half of the pictures) we normalised the white end to 97 percent using snow patches (Dawson et al. 2014). The reflectance values of the giant pandas were confirmed prior to analyses. For this, two giant pandas were photographed in Ähtäri Zoo, Finland next to photographic standards (Fig. S1). The black fur patches can be approximated as 5-10% reflectance across the visible spectrum (i.e., 400-700 nm), whereas the white patches are more variable with a reflectance range of approximately 50-80%. More generally, mammalian white pelage also varies depending on the source (Hetem et al. 2009, Dawson et al. 2014, Leblanc et al. 2016). The absolute values of fur reflectance are not crucial here; rather, it is important to normalise the images' dynamic light range across the photographs. Our choice of proxies for white and black fur produced good results in terms of balance across light levels within the dataset, as judged from the image histogram values.

Weber fractions, colour & luminance discrimination thresholds: We used custom Weber fractions of 0.05 for chromatic channels. For the luminance we used 0.20 for cat (Clark and Clark 2016), 0.22 for dog (Pretterer et al. 2004) and 0.11 for human.

Spectral sensitivities: Default spectral sensitivities for the human (trichromatic) and domestic dog (dichromatic) were used from the QCPA. In addition, we used a cat vision model using data from felids (domestic cats): lambda max = sw 454, mw 561 and cone ratio = 1:6 (Loop et al. 1987).

Spatial acuity & viewing distance: We processed the data using humans as trichromatic viewers with a visual acuity of 72 cpd, and domestic dog and cat as dichromatic viewers with visual acuities of 12 cpd and 10 cpd respectively. The viewing distance in terms of QCPA's Visual Contrast Analysis (VCA) was not scaled, but treated as from those distances from which the photographs were taken in nature (range: circa from 5 to 150 meters).

QCPA and micaToolbox settings: The protocol in the Image J using micaToolbox and QCPA framework was as follows (see below).

Converting photographs into multispectral images: We first went through each image and converted them into multispectral images. We set the white standards as previously mentioned (7% black and 63% white or 97% when snow). Then, we selected the regions of interest (ROIs): we set the scale using the size of the animal (the length was set as 1 which corresponds to approximately 1.5 m), selected the silhouette of animal (the entire outline of the giant panda) and respective background (the rectangular selection of the visual background surrounding the giant panda in approximately 1:5 animal-to-background scale). Settings were saved for further use.

Converting multispectral files into cone catch images: We re-opened the multispectral files and converted them (separately for vision models) into human, domestic dog and cat cone catch images. The negative values were removed and added with a default 0.001 value, although the results were similar if this correction was not made. Next, a luminance channel (human: lw+mw; dog&cat: mw+sw) was added into the image

stack. After this, the QCPA framework was conducted. *QCPA framework settings*: We chose the AcuityView, because of the faster processing speed, opted for the RNL-filtering, clustering RNL and used custom values of Weber fraction (chromatic 0.05, luminance 0.1). The acuity was set as 72 cpd for humans, 12 cpd for domestic dog and 10 cpd for cats. The images were rescaled to 3px/minimum resolvable angle for the whole image. The RNL filter was run with defaults: iterations 5, radius 5, falloff 3. The RNL clustering settings were also set as follows: Colour JND Threshold 9999 (i.e., chromatic thresholding disabled for luminance thresholding only), Luminance JND Threshold 1, Loops 20, Radius multiplier 3, Minimum cluster size 1000, Compare all clusters from pass 5, Stop clustering if number of clusters is below 1, Record output from pass 20.

RNL Colour map settings: The RNL colour map (Fig. S2) procedure was run with the following settings. Create colour map from ROI's: visual system Weber fractions was set 'custom', resolution (px per JND was set 4), Weber fractions were all specified as 0.05 (lw, mw, sw). Colour map plotting settings: Scale 8, Z-axis resolution (JNDs) 2, Colour options 'use colour palette', Unify colours within maps chosen, Boundary threshold 1, JND perimeter size 1, Figure border size 20, Tick length 2, Line width 2, Axis font 4, Add colour map labels chosen, Label font size 3.

QCPA's Visual Contrast Analysis: The summary table of output parameters (such as here in the Fig. S3) and their detailed explanations can be found here (see also Table S2): http://www.empiricalimaging.com/knowledge-base/running-the-qcpa-framework/

Table S2: The summary table of output parameters of QCPA's Visual Contrast Analysis and more detailed explanations. Retrieved 1.10.2021 from empirical imaging website: http://www.empiricalimaging.com/knowledge-base/running-the-qcpa-framework/

Abbreviation	Variable Name
VCA:ML	**Weighted mean of pattern luminance contrast** – *ML* (eq. 13) The mean luminance of the image (weighted by the area of each colour).
VCA:sL	**Weighted standard deviation of pattern luminance contrast** – *sL* (eq. 14). Standard deviation of the luminance of the image (weighted by the area of each colour). Higher values imply more variance in luminance values.
VCA:CVL	**Weighted CoV of pattern luminance contrast** – *CVL* (eq. 15) Coefficient of variation of luminance in the image (based on the above two values). Higher values imply more variance, while controlling for the mean value.
VCA:MDmax	**Weighted mean of pattern *Dmax* contrast** – *MDmax* (eq. 16) The mean Dmax contrast of the image (weighted by the area of each colour). Dmax is the most stimulated pairwise opponent channel (a colour channel which describes chromaticity, i.e. both hue and saturation) of each colour relative to grey (the achromatic point). Consider using the RNL saturation-based values instead for well characterised visual systems because it is a more perceptually uniform colour space.
VCA:sDmax	**Weighted standard deviation of pattern *Dmax* contrast** – *sDmax* (eq. 17). Standard deviation of the Dmax contrast of the image (weighted by the area of each colour). Higher values imply more variance in Dmax chromaticity values, and a more "colourful" image.
VCA:CVDmax	**Weighted CoV of pattern *Dmax* contrast** – *CVDmax* (eq. 18). Coefficient of variation of Dmax contrast in the image (based on the above two values). Higher values imply more variance, while controlling for the mean value.
VCA:MSsat	**Weighted mean of pattern RNL saturation contrast** – ΔSSat (eq. 19). The mean RNL saturation of the image (weighted by the area of each colour). RNL saturation is the distance (in receptor noise limited space, Vorobyev & Osorio 1998) of each colour from grey (the achromatic point). E.g. a fairly uniformly bright "red" image will have a high overall saturation, and a grey image will have a low value. Images with extremely varied colours (e.g. red, green and blue equally weighted) will also have a low mean, but high variance (see below).
VCA:sSsat	**Weighted standard deviation of pattern RNL saturation contrast** – sΔSSat (eq. 20). Standard deviation of the RNL saturation of the image (weighted by the area of each colour). Higher values imply more variance in RNL saturation values, and a more "colourful" image relative to grey. See also the VCA:MS value, which measures the internal differences between colours (i.e. colourful relative to each-other, instead of relative to grey).

VCA:CVSsat	**Weighted CoV of pattern RNL saturation** – CVSSat (eq. 21). Coefficient of variation of RNL saturation in the image (based on the above two values). Higher values imply more variance, while controlling for the mean value.
VCA:MSL	**Weighted mean of RNL luminance pattern contrast** – MSL (eq. 22). This is the average RNL luminance difference between all of the colours in the image, weighted by the area covered by each colour. Contrasts use the Siddiqi *et al.* 2004 method. Higher values imply larger luminance differences between colours in the image.
VCA:sSL	**Weighted standard deviation of RNL luminance pattern contrast** – sΔSL (eq. 23). The standard deviation of RNL luminance differences between all of the colours in the image, weighted by the area covered by each colour. Higher values imply larger variance in internal luminance differences, e.g. lots of similar and dissimilar luminance differences.
VCA:CVSL	**Weighted CoV of RNL luminance pattern contrast** – CVSL(eq. 24). Coefficient of variation of internal RNL luminance differences in the image (based on the above two values). Higher values imply more variance, while controlling for the mean value.
VCA:MS	**Weighted mean of pattern RNL chromaticity contrast** – MΔS (eq. 25). This is the average colour difference between all of the colours in the image, weighted by the area covered by each colour. Contrasts use the Vorobyev & Osorio 1998 Receptor Noise Limited method. Higher values imply larger internal colour differences between colours in the image. Therefore an image with lots of different colours will have a high value, irrespective of how far those colours are from the grey point. E.g. if the entire image is fairly uniformly bright “red” this value will be low, because all of the internal colours are similar. However, the saturation of the image (e.g. MSsat above) will be high.
VCA:sS	**Weighted standard deviation of pattern RNL chromaticity contrast** – $s\Delta S$ (eq. 26). The standard deviation of RNL colour differences between all of the colours in the image, weighted by the area covered by each colour. Higher values imply larger variance in internal colour differences, e.g. lots of similar and dissimilar colours.
VCA:CVS	**Weighted CoV of pattern RNL chromaticity contrast** – CVΔS (eq. 27). Coefficient of variation of internal RNL colour differences in the image (based on the above two values). Higher values imply more variance, while controlling for the mean value.

Table S3: Summary of giant panda camouflage analyses with respect to ROI ('region of interest') and vision model (vision). Linear mixed effects model (LMER) analyses of the animal-to-background clustering results were used, which ANOVA table with Satterthwaite's method is reported here. The ROI was set as panda, background and the whole image, and were modelled through human, canine and felid vision models. Dependent variables: number of clusters is the number of receptor-noise limited visual clusters formed, cluster luminance is the luminance of the respective visual clusters, cluster size is the area of clusters in pixels, relative coverage is the relative size of clusters within the ROI and edge disruption is the Gabor filtering to quantify ratio of false edges that run orthogonal to animal's true outline (also known as GabRat).

Subject	Term	SS	MS	DF	F	P
Number of clusters	ROI	3.56	1.78	2	0.337	0.713
	Vision	952.31	476.15	2	90.226	<0.001
	ROI * Vision	2.63	0.66	4	0.124	0.973
Cluster luminance	ROI	0.135	0.067	2	0.864	0.421
	Vision	0.057	0.028	2	0.367	0.692
	ROI * Vision	0.027	0.006	4	0.086	0.986
Cluster size	ROI	1.759e+12	8.796e+11	2	212.785	<0.001
	Vision	2.650e+11	1.325e+11	2	32.059	<0.001
	ROI * Vision	1.784e+11	1.787+11	4	10.810	<0.001
Relative coverage	ROI	923.5	461.7	2	1.925	0.146
	Vision	23070.6	11535.3	2	48.105	<0.001
	ROI * Vision	23.1	5.8	4	0.024	0.998
Edge disruption	Distance	1.166	1.166	1	365.124	<0.001
	Vision	0.041	0.020	2	6.481	0.001
	Distance * Vision	0.254	0.127	2	39.770	<0.001

Table S4: Giant panda background matching: number of clusters formed by the QCPA analysis. The clustering method uses spatial filtering to test which visual elements cluster together in the image performed using acuity-corrected cone-catch images (canine vision – dog 12 cpd and felid vision – cat 10 cpd, and the trichromatic human vision 72 cpd). Panda ID was included as random factor (variance = 0.312 s.d. = 0.558) in linear mixed effects model (lmer) in R. Full model is reported here.

Subject	Estimate	s.e.	t-value	P
ClusterID				
(Intercept)°	5.351	0.240	22.300	<0.001
ROI Panda	-0.178	0.273	-0.652	0.514
ROI Background	-0.347	0.289	-1.201	0.230
VISION Cat	-2.167	0.324	-6.673	<0.001
VISION Dog	-2.469	0.340	-7.253	<0.001
ROI Background * VISION Cat	0.178	0.460	0.388	0.698
ROI Panda * VISION Cat	0.324	0.477	0.680	0.497
ROI Background * VISION Dog	0.110	0.483	0.229	0.819
ROI Panda * VISION Dog	0.205	0.500	0.403	0.687

°Intercept includes factor level(s): ROI [Whole image], vision [Human].

Table S5: Giant panda background matching: luminance distribution predicted by the QCPA analysis. The clustering method uses spatial filtering to test which visual elements cluster together in the image performed using acuity-corrected cone-catch images (canine vision – dog 12 cpd and felid vision – cat 10 cpd, and the trichromatic human vision 72 cpd). Panda ID was included as random factor (variance = 0.004 s.d. = 0.070) in linear mixed effects model (lmer) in R. Full model is reported here.

Subject	Estimate	s.e.	t-value	P
Luminance				
(Intercept)°	0.326	0.029	11.059	<0.001
ROI Panda	-0.014	0.033	-0.438	0.661
ROI Background	-0.045	0.035	-1.292	0.197
VISION Cat	-0.015	0.039	-0.398	0.691
VISION Dog	-0.006	0.041	0.168	0.866
ROI Background * VISION Cat	0.014	0.056	0.261	0.794
ROI Panda * VISION Cat	0.029	0.058	0.501	0.616
ROI Background * VISION Dog	0.021	0.058	0.369	0.713
ROI Panda * VISION Dog	0.015	0.060	0.258	0.797

°Intercept includes factor level(s): ROI [Whole image], vision [Human].

Table S6: Giant panda background matching: cluster size (in pixel area) estimated by the QCPA analysis. The clustering method uses spatial filtering to test which visual elements cluster together in the image performed using acuity-corrected cone-catch images (canine vision – dog 12 cpd and felid vision – cat 10 cpd, and the trichromatic human vision 72 cpd). Panda ID was included as random factor (variance = 6.945e+07, s.d. = 0.8334) in linear mixed effects model (lmer) in R. Full model is reported here.

Subject	Estimate	s.e.	t-value	P
Cluster size				
(Intercept)°	73447.0	5779.0	12.709	<0.001
ROI Panda	-40147.7	7660.2	-5.241	<0.001
ROI Background	-71904.7	8089.9	-8.888	<0.001
VISION Cat	64007.3	9085.0	7.045	<0.001
VISION Dog	84398.6	9519.0	8.866	<0.001
ROI Background * VISION Cat	-37002.8	12886.0	-2.872	<0.001
ROI Panda * VISION Cat	-61956.4	13347.0	-4.642	<0.001
ROI Background * VISION Dog	-47624.4	13527.7	-3.521	<0.001
ROI Panda * VISION Dog	-82419.1	13999.2	-5.887	<0.001

°Intercept includes factor level(s): ROI [Whole image], vision [Human].

Table S7: Giant panda background matching: cluster relative coverage (in pixel area) estimated by the QCPA analysis. The clustering method uses spatial filtering to test which visual elements cluster together in the image performed using acuity-corrected cone-catch images (canine vision – dog 12 cpd and felid vision – cat 10 cpd, and the trichromatic human vision 72 cpd). Panda ID was included as random factor (variance = 11.04, s.d. = 3.32) in linear mixed effects model (lmer) in R. Full model is reported here.

Subject	Estimate	s.e.	t-value	P
Relative coverage				
(Intercept)°	10.691	1.551	6.892	<0.001
ROI Panda	0.433	1.845	0.235	0.814
ROI Background	3.027	1.949	1.553	0.121
VISION Cat	9.514	2.189	4.346	<0.001
VISION Dog	12.409	2.294	5.409	<0.001
ROI Background * VISION Cat	-0.433	3.103	-0.140	0.889
ROI Panda * VISION Cat	-0.987	3.215	-0.307	0.759
ROI Background * VISION Dog	-0.127	3.258	-0.039	0.969
ROI Panda * VISION Dog	-0.470	3.372	-0.139	0.889

°Intercept includes factor level(s): ROI [Whole image], vision [Human].

Table S8: Giant panda edge disruption. The method uses Gabor filtering to quantify ratio of false edges that run orthogonal to animal's true outline. Linear mixed effects model (LMER) analyses of the achromatic edge contrast (GabRat) with respect to distance and its relation to predator vision. The edge disruption was modelled through three vision models and their respective visual acuities: dichromatic cat (10 cpd) and dog (12 cpd) and trichromatic human vision (72 cpd). Panda ID was included as random factor (variance = 0.002 s.d. = 0.04) in the model.

Subject	Estimate	s.e.	t-value	P
Edge disruption (GabRat)				
(Intercept)°	3.261e-01	1.333e-02	24.452	<0.001
Distance	6.374e-04	3.985e-05	15.995	<0.001
VISION Dog	2.585e-04	8.546e-03	0.030	0.975
VISION Human	-2.652e-02	8.546e-03	-3.103	0.002
Distance * VISION Dog	1.128e-04	5.636e-05	-2.001	0.046
Distance * VISION Human	-4.806e-04	5.636e-05	-8.527	<0.001

°Intercept includes factor level(s): Vision [Cat].

Figure S1: Calibration results of the photographic standards and giant panda pelage patches. A) Two captive giant pandas were photographed in Ähtäri Zoo, Finland next to a set of white, grey and black photographic standards. Several photos were taken from both individuals from different angles to determine giant panda fur reflectance in comparison to standards with known reflectance values. B) With the aid of this information (white pelage mean = 62.9, s.d. =16.24; black pelage, mean = 6.96, s.d. = 3.74), we normalised the white balance (i.e., the dynamic light range in our photographic data as there were no other consistent features in the photos taken in the wild than the giant pandas themselves.

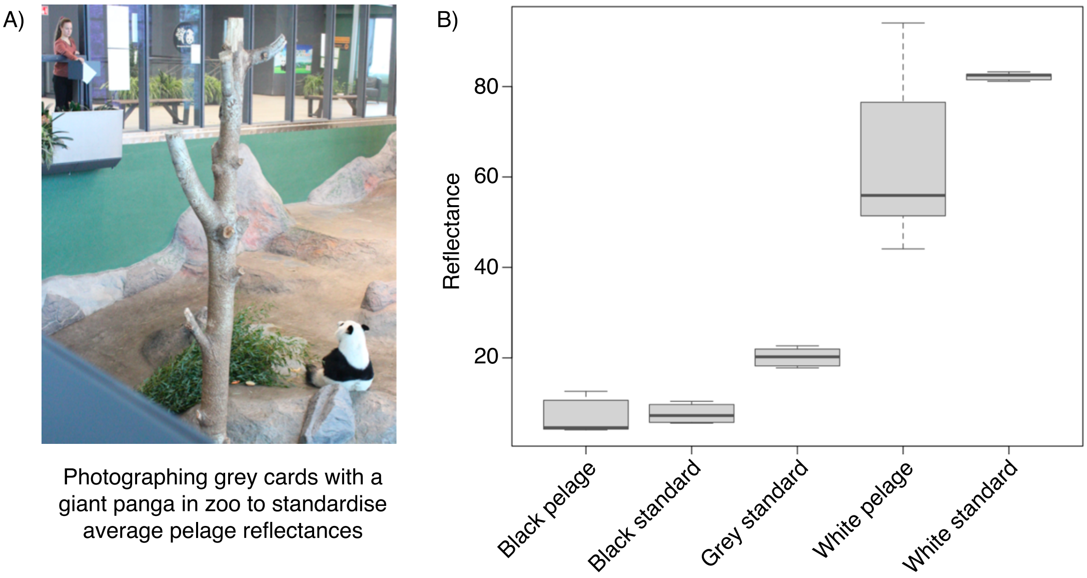

Figure S2: Similarity to background measured as overlap in colour space. A) The chromaticity diagram using human vision shows that 50 % of all colours within images were perceptually similar between the giant panda and its background. The diagram axes refer to perceived chromaticity similarity in red-green and blue-yellow axes. B) We also compared the similarity-to-background index with other species. The least similar species to their respective backgrounds are those considered as being aposematic, whereas the other end of the spectrum consists of the camouflaged animals. The giant panda falls at the middle of this 'conspicuity spectrum', and among other species that are traditionally considered as camouflaged, which suggests that giant panda pelage coloration does not stand out against the background more than a concealed species would. All inset pictures (backgrounds removed): Wikimedia commons open access license (CC BY-SA 3.0).

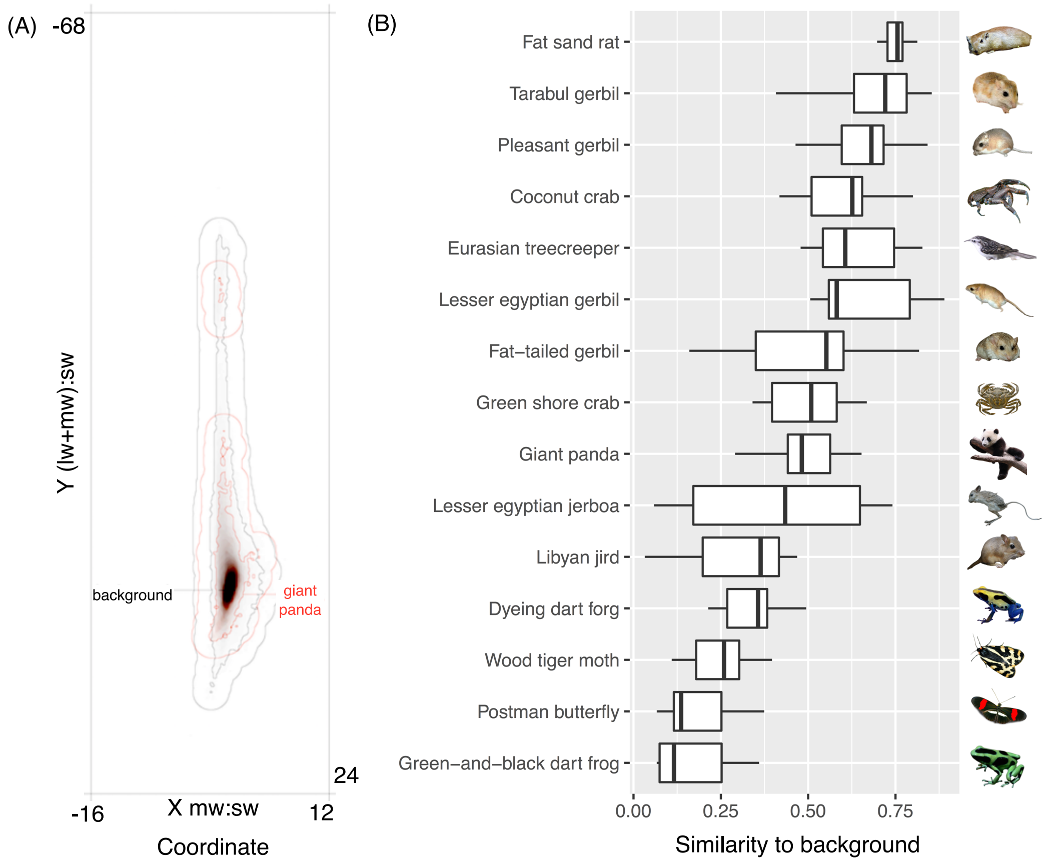

Figure S3: Visual Contrast Analysis - VCA. The figure shows the relationship between the measured ROI (background, panda, whole image) and vision model used (domestic dog, cat or human). For the details of the colour metrics refer to (van den Berg et al. 2020) or summary table output parameters (Table S2) from empirical imaging website (http://www.empiricalimaging.com/knowledge-base/running-the-qcpa-framework/).

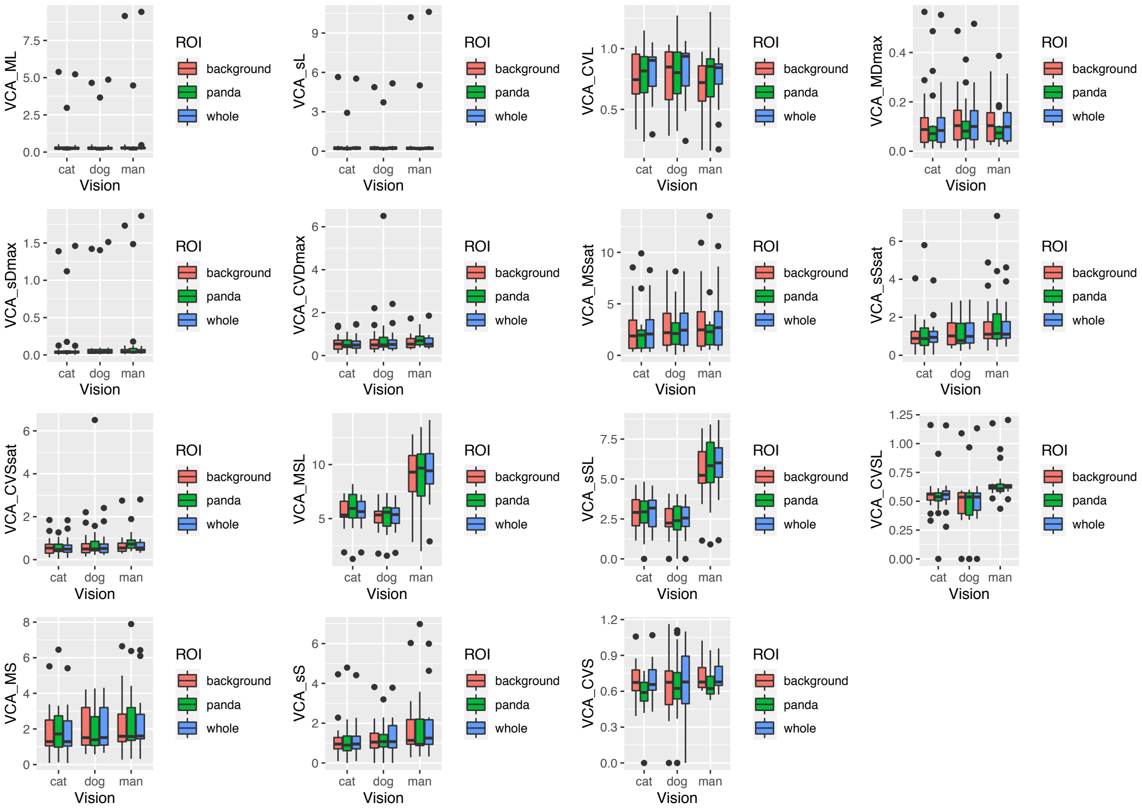

Figure S4: Patch clustering divided by season (summer vs. winter). The clustering method uses spatial filtering to test which visual elements cluster together in the image performed using acuity-corrected cone-catch images (canine vision – dog 12 cpd and felid vision – cat 10 cpd, and the trichromatic human vision 72 cpd). Clustering results are presented for number of clusters formed (A, B), cluster luminance distribution (B, C), cluster size as area measured in pixels (D, E), and the relative coverage of clusters within the ROI (G, H).

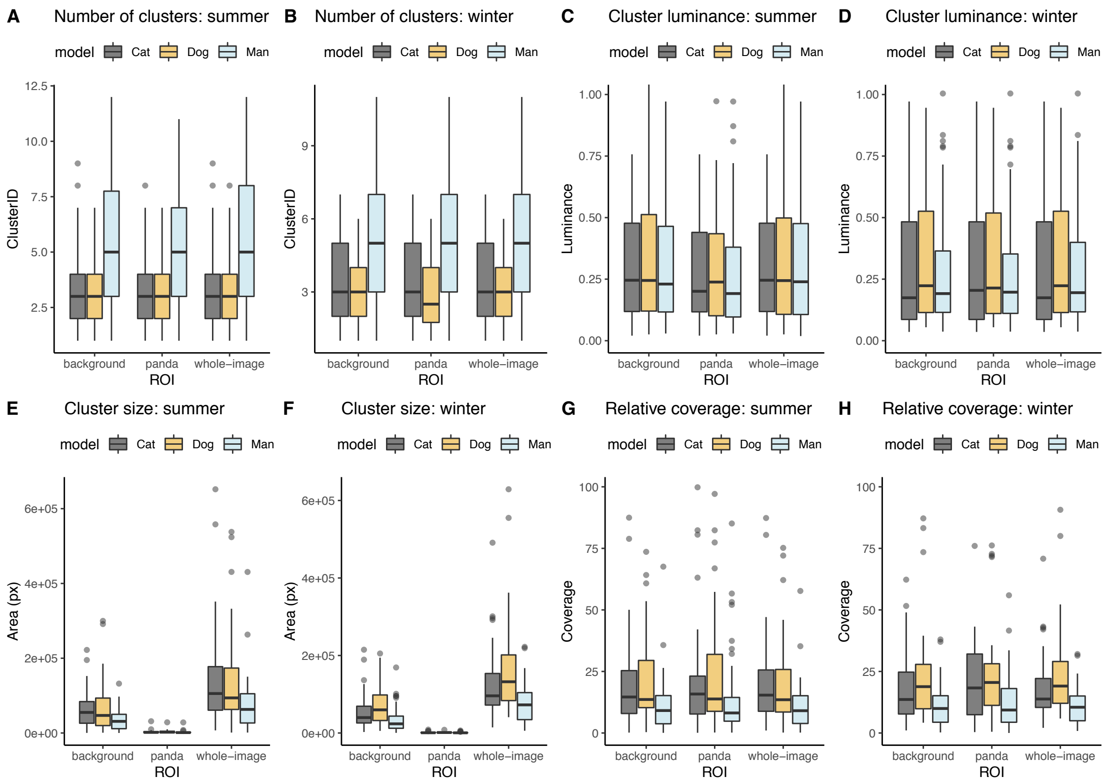

Figure S5: Giant panda edge disruption: No seasonal differences. The method uses Gabor filtering to quantify the ratio of false edges that run orthogonal to the animal's true outline. Edge disruption was modelled through three visual models and their respective acuities: dichromatic felid (cat - 10 cpd) and canine (dog -12 cpd), as well as trichromatic human vision (72 cpd). The boxplot shows minimum and maximum (whiskers), median horizontal line and the interquartile range of the simulated data. There was no marked difference between winter (with snow) and summer conditions on edge disruption within dataset.

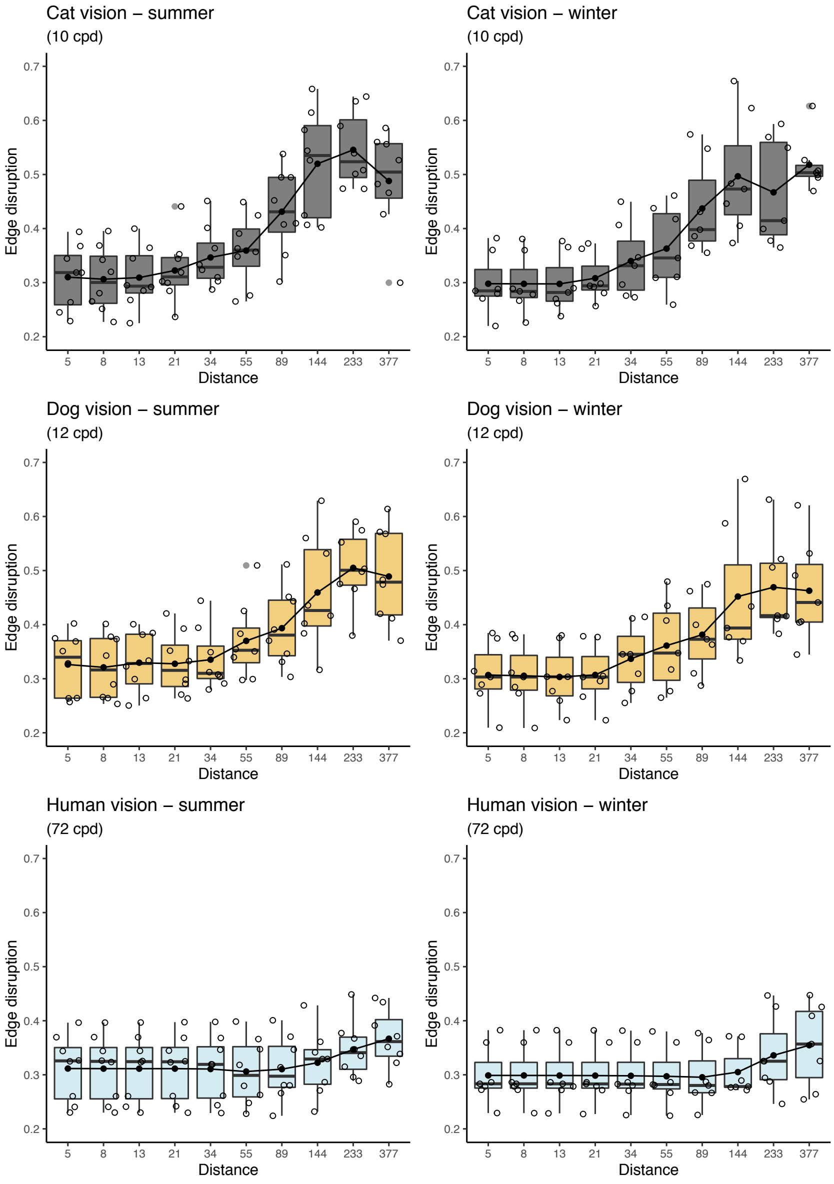

References:

van den Berg, C. P., J. Troscianko, J. A. Endler, N. J. Marshall, and K. L. Cheney. 2020. Quantitative Colour Pattern Analysis (QCPA): A Comprehensive Framework for the Analysis of Colour Patterns in Nature. Methods in Ecology and Evolution 11:316–332.

Caves, E. M., N. C. Brandley, and S. Johnsen. 2018. Visual Acuity and the Evolution of Signals. Trends in Ecology & Evolution 33:1–15.

Clark, D. L., and R. A. Clark. 2016. Neutral point testing of color vision in the domestic cat. Experimental Eye Research 153:23–26.

Lind, O., I. Milton, E. Andersson, P. Jensen, and L. S. V. Roth. 2017. High visual acuity revealed in dogs. PLoS ONE 12:1–12.

Loop, M. S., L. C. Millican, and S. R. Thomas. 1987. Photopic spectral sensitivity of the cat. Journal of Physiology 382:537–553.

Pretterer, G., H. Bubna-Littitz, G. Windischbauer, C. Gabler, and U. Griebel. 2004. Brightness discrimination in the dog. Journal of Vision 4:241–249.

Troscianko, J., and M. Stevens. 2015. Image Calibration and Analysis Toolbox - a free software suite for objectively measuring reflectance, colour and pattern. Methods in Ecology and Evolution 6:1320–1331.
